# Using Bosentan to Treat Paraquat Poisoning-Induced Acute Lung Injury in Rats

**DOI:** 10.1371/journal.pone.0075943

**Published:** 2013-10-14

**Authors:** Zhongchen Zhang, Xiangdong Jian, Wei Zhang, Jieru Wang, Qian Zhou

**Affiliations:** Department of Poisoning and Occupational Disease, Qilu Hospital of Shandong University, Jinan, Shandong, China; University of Colorado, United States of America

## Abstract

**Background:**

Paraquat poisoning is well known for causing multiple organ function failure (MODS) and high mortality. Acute lung injury and advanced pulmonary fibrosis are the most serious complications. Bosentan is a dual endothelin receptor antagonist. It plays an important role in treating PF. There is no related literature on the use of bosentan therapy for paraquat poisoning.

**Objective:**

To study the use of bosentan to treat acute lung injury and pulmonary fibrosis as induced by paraquat.

**Method:**

A total of 120 adult Wister male rats were randomly assigned to three groups: the paraquat poisoning group (rats were intragastrically administered with paraquat at 50 mg/kg body weight once at the beginning); the bosentan therapy group (rats were administered bosentan at 100 mg/kg body weight by intragastric administration half an hour after paraquat was administered, then the same dose was administered once a day); and a control group (rats were administered intragastric physiological saline). On the 3rd, 7th, 14th, and 21st days following paraquat exposure, rats were sacrificed, and samples of lung tissue and venous blood were collected. The levels of transforming growth factor-β1 (TGF-β1), endothelin-1 (ET-1), and hydroxyproline (HYP) in the plasma and lung homogenate were determined. Optical and electronic microscopes were used to examine pathological changes.

**Result:**

The TGF-β1, ET-1, and HYP of the paraquat poisoning group were significantly higher than in the control group, and they were significantly lower in the 21st day therapy group than in the paraquat poisoning group on the same day. Under the optical and electronic microscopes, lung tissue damage was observed to be more severe but was then reduced after bosentan was administered.

**Conclusion:**

Bosentan can reduce inflammation factor release. It has a therapeutic effect on acute lung injury as induced by paraquat.

## Introduction

Paraquat (PQ) (1,1′-dimethyl-4,4′-bipyridinium dichloride) is a non-selective contact herbicide that has been used widely in many countries since the 1960s. It is highly toxic to humans and is associated with high mortality, mainly as a consequence of respiratory failure. The most common poisoning route is by ingestion of the concentrated solution, either intentional or accidental. On rare occasions, there are also cases of intoxication by way of dermal exposure or intravenous injections. Dermal exposure reportedly results in severe PQ poisoning, especially in the presence of pre-existing skin lesions. Intravenous PQ poisoning is rare [1], and the clinical presentation and prognosis of such a scenario would be quite different from that of oral ingestion.

Plasma PQ levels peak at two hr after PQ ingestion and decrease rapidly. PQ is eliminated by the kidney in urine [2]. Severe PQ poisoning is characterized by multiple-organ failure, which mainly involves the lungs, kidneys, liver, heart and also the nervous system. The lung is a major target organ in paraquat poisoning, and respiratory failure from lung injury is the most common cause of death [3] [4]. The exact mechanism of PQ poisoning is currently unknown. Because of a lack of effective antidotes, conventional supportive treatments are poor and have a high mortality rate. Many scholars are devoted to this subject [5] [6]. Pulse therapy with cyclophosphamide and methylprednisolone has significantly prevented pulmonary fibrosis (PF) in rats [7].

Endothelin (ET) is an endothelium-derived 21-residue vasoconstrictor peptide. It was isolated by a Japanese scholar in 1988, and has been shown to be one of the most potent known vasoconstrictors [8]. The ETs have three distinct isoforms, namely ET-1, ET-2, and ET-3. ET-1 is the most abundant isoform and the best characterized in vivo, and it is the only one that is constitutively released by the vascular endothelium. In addition, ET-1 is also produced by smooth muscle cells, macrophages, airway epithelium, and alveolar epithelial cells. ET-1 is released following various injurious stimuli, including shear stress, thrombin, angiotensin II, cytokines, and free radicals [9]. ET-1 plays a central role in lung fibrosis [10], and it has been shown to induce fibroblast proliferation, differentiation, contraction, and collagen synthesis. It also independently promotes fibroblast resistance to apoptosis through signaling pathways [11] [12]. The plasma ET levels in patients with MODS caused by acute PQ poisoning were much higher than those of the controls [13] [14].

Bosentan is a dual endothelin-receptor antagonist that can be taken orally, and in patients with severe PH, it can improve exercise capacity, hemodynamics and quality of life [15] [16]. Bosentan application to bleomycin-treated rats resulted in significantly higher exercise capacity as a result of improvements in PH and PF and improvements in their quality of life [17] [18]. The present data suggests a therapeutic utility for bosentan, particularly in treating the early stages of chronic inflammatory airway diseases. The present study was designed to evaluate the effects of bosentan as a PQ poisoning treatment.

## Materials and Methods

### Reagents

Twenty percent imported PQ solution was purchased from Syngenta Nantong Crop Protection Co. Ltd, Nantong, Jiangsu, China. The reagents and instruments were as follows: detection kits for transforming growth factor-β1 (TGF-β1), endothelin 1 (ET-1) and hydroxyproline (HYP) (all from Nanjing Jiancheng Biological Engineering Institute, China). Bosentan was provided by Actelion Pharmaceuticals Ltd., Switzerland (lot number 704001).

### Animals

Animal experiments were performed according to international guidelines. This study was approved by the Institutional Animal Care and Use Committee of Shandong University.

One hundred twenty adult male Wister rats (230–250 g) were obtained from the Experimental Animal Center of Shandong University, lot number SCXK(LU)20030004. All of them were administered a standard rat diet and tap water ad libitum, and maintained under the following controlled conditions: 12 hours light/dark cycle, (22±2)°C room temperature, and 50%–60% humidity for at least 1 week before the experiment. The rats were randomly divided into three groups of forty rats each: the paraquat poisoning group, the bosentan therapy group and the control group.

### Animal models and tissue sampling

The rats in the PQ and bosentan therapy groups were administered intragastric doses of 50 mg/kg PQ once at the beginning. In the bosentan therapy group, the rats were intragastrically administered with 100 mg/kg bosentan half an hour after PQ exposure, and then once a day. The rats in the PQ group intragastrically received equal amounts of sterile normal saline once a day. The rats in the control group were simultaneously administered equal amounts of sterile normal saline once a day.

On the 3rd, 7th, 14th, and 21st day after PQ administration, ten rats were selected from the PQ, bosentan therapy and control groups. These rats were anesthetized with an intraperitoneal injection of 4% chloral hydrate and immediately sacrificed. After a blunt dissection of each inferior vena cava, approximately 4 ml of blood was collected and placed in a disposable plastic tube. The blood samples were centrifuged at 5000 r/min for 5 minutes, and the serum was stored at −70°C to detect ET-1 and TGF-β1. To distinguish the rats' left and right lungs, each left lung was placed in a frozen pipette and stored in a −70°C liquid nitrogen freezer to detect the ET-1,TGF-β1 and HYP. The lower right lung was immersed in 10% buffered formaldehyde and then embedded in paraffin. The lung lobes were subsequently separated and sectioned sagittally into 5-µm-thick sections, then stained with hematoxylin, eosin (HE) and Masson's trichrome. The sections were examined by light microscopy and assessed for the presence of hemorrhage, intra-alveolar edema and fibrosis.

Samples were immediately placed in cold 2.5% glutaraldehyde in phosphate buffer to pH 7.2. They were then postfixed in 1% osmic acid, similarly buffered for 1 hour, dehydrated in a graded series of acetone, and embedded in Epon. In this fixative, they were minced into small (approximately 1 mm cubes) fragments and transferred to fresh fixative and fixed for an additional 2 hours. Ultrathin sections were stained with uranyl acetate and lead citrate for electron microscopic study. These sections were examined and photographed in a Hitachi H-800 (Hitachi, Ltd. Tokyo, Japan) transmission electron microscope.

### ET-1 and TGF-β1 assays

ET-1 and TGF-β1 were quantified by ELISA kits (Nanjing Jiancheng Biological Engineering Institute, China). Assays were performed according to the manufacturer's instructions.

### Hydroxyproline determination

The HYP content was determined after acid hydrolysis of the lung tissue with 12 N HCl at 100°C for 20 min. The data were expressed as micrograms of HYP per milligram of lung tissue (µg/mg).

### Statistical analysis

Normally distributed data are presented as the mean ± standard deviation (SD). Using the SPSS 13.0 software package, a statistical analysis was used to compare mean values from three groups by independent samples t-test. A P<0.05 was considered significant.

## Results

### Animal model characteristics

All the rats were irritable after intragastric PQ administration, and their body weights decreased over the next few days. A wheezing sound and nasal hemorrhage was observed in the individuals. No significant difference was noted in the weights of the bosentan therapy group and the control.

### Dynamic changes in the ET-1 and TGF-β1 of the serum and lung, and the HYP in the lung

The dynamic changes of ET-1 and TGF-β1 in the serum and lung, and the HYP in the lung are detailed in Tables 1–4. In the serum and lung of the PQ group rats, the ET-1 and TGF-β1 content significantly increased (all P<0.05) compared to those of the control group. The ET-1 and TGF-β1 content gradually became higher in the serum and lungs, and reached their highest points on the 21st day. After bosentan administration, the ET-1 and TGF-β1 content of the serum and lungs of the bosentan therapy group rats gradually decreased and was markedly lower than in the PQ group on the 21st day (P<0.05). The HYP of the PQ group increased from the 3rd day after PQ exposure (all P<0.05), and the bosentan treatment delayed the increase and was markedly lower than that of the PQ group on the 21st day (P<0.05) (Table 5).

**Table 1:** Dynamic change of ET-1 in the serum from the three groups (mean±SD, ng/L).

Groups	PQ group	Bosentan therapy group	Control group
Day 3	192.99±8.72	190.03±11.15	165.87±1.79
Day 7	201.77±6.40	184.61±12.42	165.96±1.95
Day 14	209.54±8.52	172.16±7.75	166.20±2.08
Day 21	214.90±13.57	168.33±8.91	166.76±2.09

**Table 2:** Dynamic change of ET-1 in the lungs of the three groups (mean±SD, ng/L).

Groups	PQ group	Bosentan therapy group	Control group
Day 3	154.44±13.73	154.31±12.91	123.92±8.07
Day 7	160.42±13.92	147.27±18.18	124.85±8.01
Day 14	199.18±14.56	131.77±9.48	124.87±8.13
Day 21	216.44±11.27	127.44±13.30	125.40±8.37

**Table 3:** Dynamic change of TGF-β1 in serum from the three groups (mean±SD, ng/L).

Groups	PQ group	Bosentan therapy group	Control group
Day 3	92.77±6.01	86.18±9.79	73.76±8.59
Day 7	97.50±8.59	82.88±7.91	73.88±8.61
Day 14	106.89±9.63	80.84±7.72	74.12±8.67
Day 21	112.30±9.12	76.70±9.76	74.19±8.79

**Table 4:** Dynamic change of TGF-β1 in the lungs of the three groups (mean±SD, ng/L).

Groups	PQ group	Bosentan therapy group	Control group
Day 3	87.22±12.74	82.97±14.28	69.89±7.01
Day 7	89.33±12.88	77.41±9.10	70.01±7.05
Day 14	92.85±14.02	74.20±7.26	70.10±7.17
Day 21	105.74±12.69	70.44±9.20	70.07±7.24

**Table 5:** Dynamic change of HYP in the lungs of the three groups (mean±SD, µg/mg).

Groups	PQ group	Bosentan therapy group	Control group
Day 3	1.12±0.20	1.06±0.19	0.52±0.08
Day 7	1.15±0.13	0.97±0.20	0.57±0.09
Day 14	1.20±0.17	0.81±0.10	0.65±0.09
Day 21	1.24±0.14	0.77±0.09	0.69±0.12

### Histomorphology

#### Macroscopic changes

The lung tissues of the PQ group were swollen, and punctate hemorrhages could be observed in some of them.

#### Optical Microscopy

**Figure 1:**
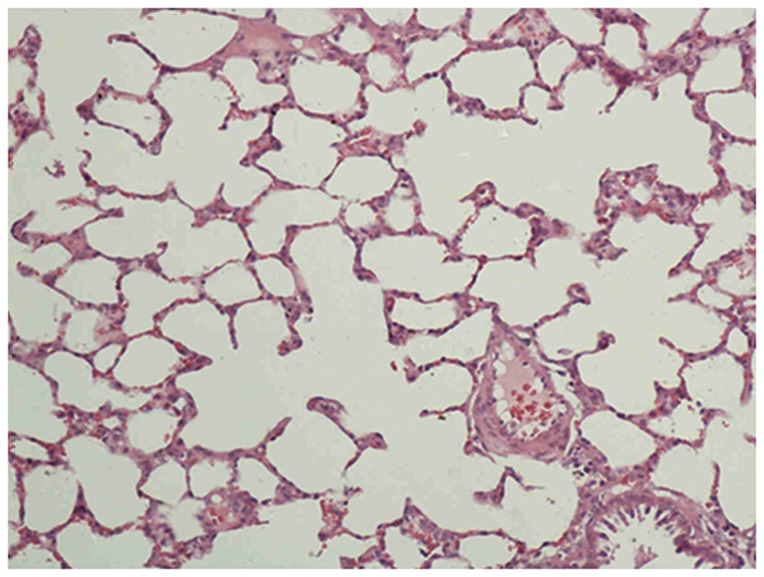
HE stain of control group (×200). Lung tissue is normal. The alveolar space has a normal interstitium structure and no effusion.

**Figure 2:**
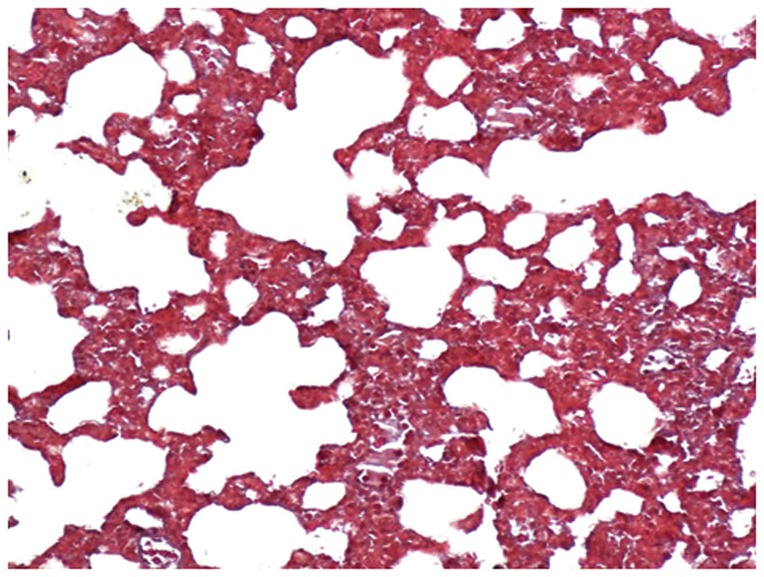
Masson stain of control group (×200). Lung tissue is normal. The alveolar space has a normal interstitium structure, and no effusion.

**Figure 3:**
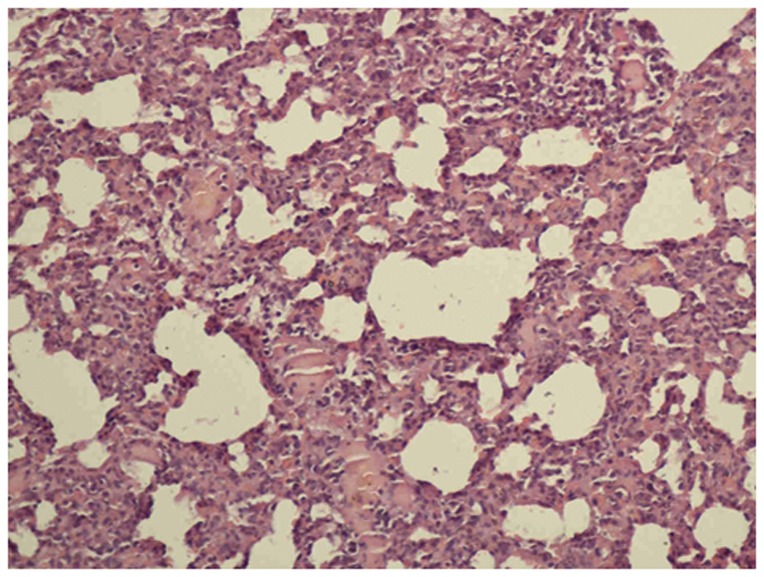
HE stain of PQ group (×200). The inflammatory process with marked infiltration of leukocytes into interstitial and alveolar spaces, edema, alveolar distortion, and thickening of the alveolar wall.

**Figure 4:**
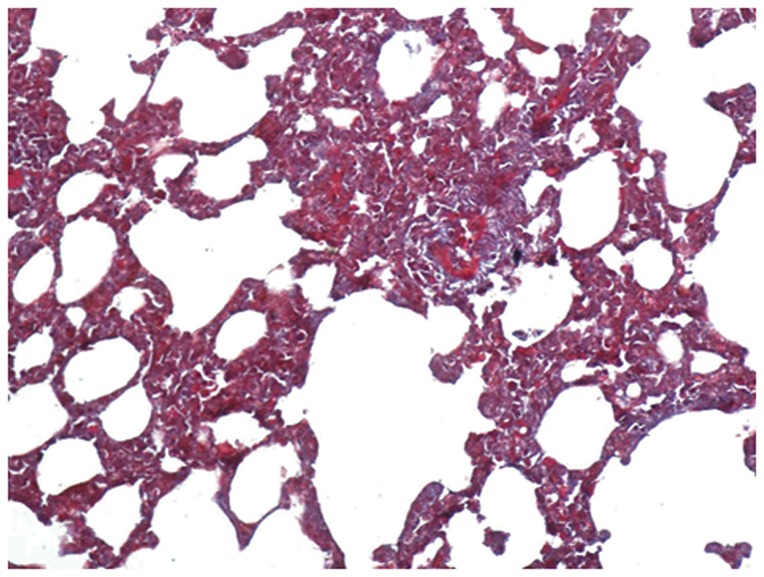
Masson stain of PQ group (×200). The alveolar wall was significantly thickened, there was evidence of phoroblast hyperplasia and fibroglia fibrils, which were obviously hyperplastic.

**Figure 5:**
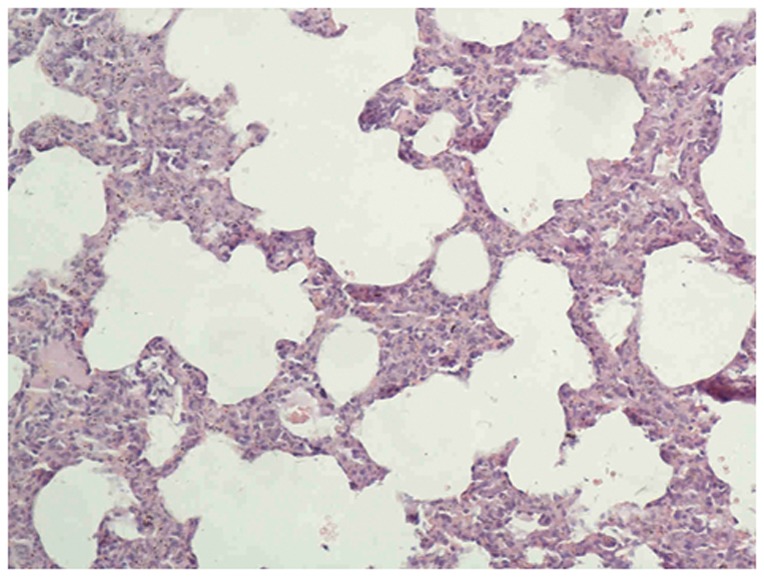
HE stain of bosentan group (×200). The damage is improved more than that of the PQ group, and less inflammatory cell infiltration were found in the interstitial lung and alveoli, interstitial edema and alveolar hemorrhage were ameliorated.

**Figure 6:**
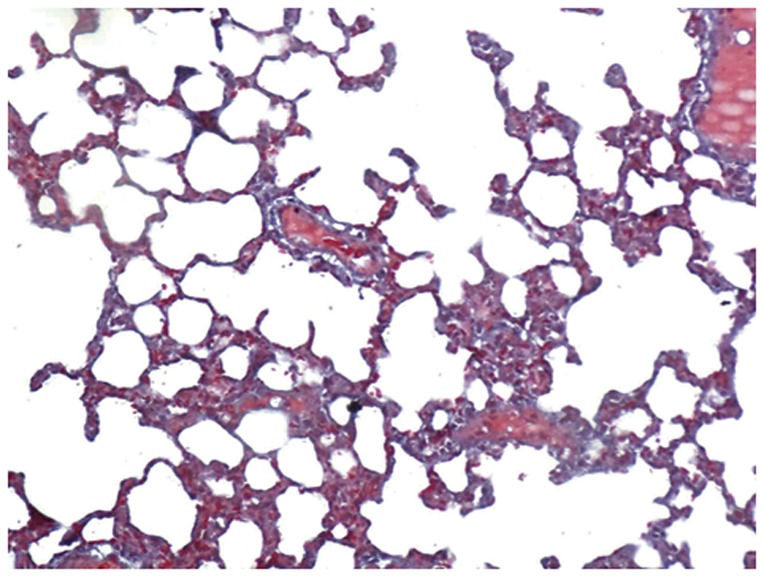
Masson stain of bosentan group (×200). The damage is improved relative to the PQ group. Alveolus interstitium was thinner and few phoroblast was observed.

The lungs of the control group were characterized by an alveolar space with normal interstitium structure (Figures 1–2). In the PQ group, substantial inflammatory cell infiltration such as lymphocytes were found in the interstitial lung and alveoli, with interstitial edema, diffuse hemorrhage, and thickened alveolus interstitium, and the condition was even more serious under longer exposure times. The alveolar wall was significantly thickened, and there was evidence of phoroblast hyperplasia and the fibroglia fibrils were obviously hyperplastic (Figure 3–4). In the bosentan therapy group, less inflammatory cell infiltration were found in the interstitial lung and alveoli, interstitial edema and alveolar hemorrhage were ameliorated. Alveolus interstitium was thinner and less phoroblast was observed (Figure 5–6).

#### Ultrastructure changes

**Figure 7:**
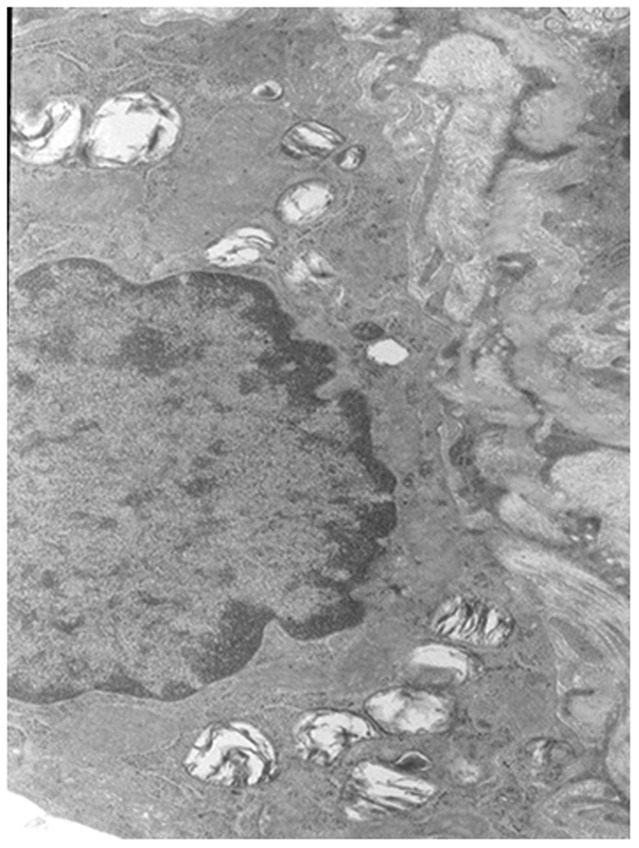
The Type II alveolar epithelial cells of the control group (×8000). The Type II alveolar epithelial cells have a normal structure.

The Type II alveolar epithelial cells of the control group showed a normal structure, and their nuclei were large and ovoid with integral and smooth membranes. The mitochondrial cristae were limpid and integral. Abundant lamellar bodies could be observed with different stages of development (Figure 7).

**Figure 8:**
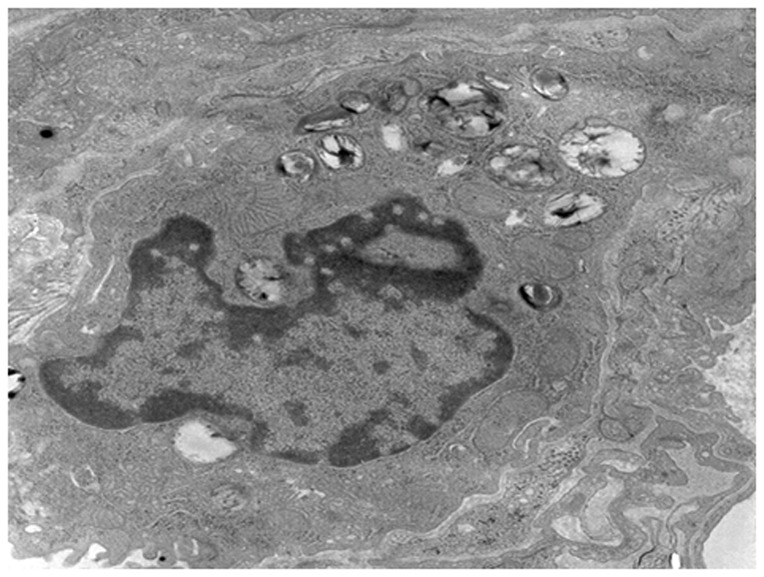
The Type II alveolar epithelial cells from the PQ group (×8000). The nuclei were collapsed and contained dense heterochromatin; the endoplasmic reticulum tended to be vesiculated, the basement membranes were absent or disrupted, the mitochondria was vacuolated and the number of lamellar bodies clearly decreased.

**Figure 9:**
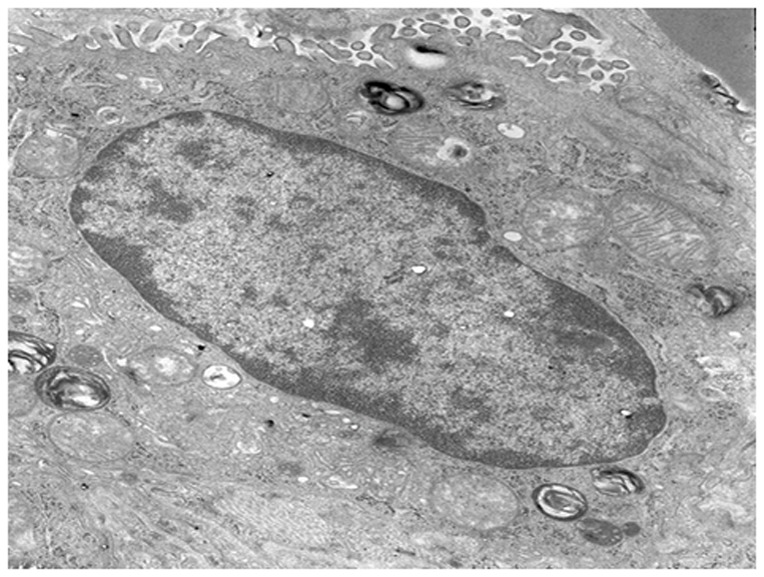
The Type II alveolar epithelial cells in the bosentan group (×8000). The damage was ameliorated to some degree relative to the PQ group. Their nuclei membrane and the endoplasmic reticulum were integral and smooth, the mitochondrial cristae were limpid but still a litter disorder, vacuolation of the lamellar bodies were ameliorated, a few mature lamellar bodies were observed.

The Type II alveolar epithelial cells of the PQ group were characterized by integral nuclei membranes, disrupted mitochondrial cristae, swelling endoplasmic reticulum (ER), vacuolation of the lamellar bodies, and immature lamellar bodies. On the 21st day, the nuclei were collapsing and contained dense heterochromatin; the endoplasmic reticulum in these cells tended to be vesiculated, the basement membranes were absent or disrupted, the mitochondria were vacuolated and the number of lamellar bodies clearly decreased (Figure 8). In comparison to the PQ group, the degenerative changes consisting of mitochondrial swelling, vacuolation of lamellar bodies, and disruption of the endoplasmic reticulum, which were ameliorated in the bosentan group. Their nuclei membrane and the endoplasmic reticulum were integral and smooth, the mitochondrial cristae were limpid but still a litter disorder, vacuolation of the lamellar bodies were ameliorated, a few mature lamellar bodies were observed (Figure 9).

## Discussion

The first two cases of paraquat poisoning were reported in 1966 [19], then PQ poisoning cases were reported all over the world. Although many investigations have been conducted, the poisoning mechanism is still unknown, no effectve antidotes exist, and mortality is high.

Over the past few years, cytokine research has brought new ideas to PQ therapy. TGF-β1 is a multifunction cytokine; it can stimulate the proliferation of fibroblasts and myofibroblasts, synthesize extracellular matrix and suppress collagen degradation, promote collagen deposition, and it may then play a pivotal role in the pathogenesis of PF [20]. One domestic study found that the level of caveolin-1 decreased in rats after PQ exposure [21]. Caveolin-1 notably downregulates transforming growth factor (TGF)-β signal transduction by modulating TGF-β1 receptor gene expression, preventing Smad2 phosphorylation, and/or mediating TGF-β receptor turnover [22].

ET-1 also plays a central role in lung fibrosis [10]. ET-1 has been shown to induce fibroblast proliferation, differentiation, contraction, and collagen synthesis. It also independently promotes fibroblast resistance to apoptosis through signaling pathways [11] [12]. The levels of plasma ET in patients with MODS caused by acute PQ poisoning were much higher than in the controls [13] [14]. An ETA receptor antagonist was shown to inhibit the development of paraquat-induced PF, confirming the importance of ET-1 in mediating PF in a rat experiment [23].

There are three distinct isoforms endothelins, namely ET-1, ET-2, and ET-3 and two endothelin receptors, namely ETA-R and ETB-R. ETA-R mainly resides in smooth muscle cells, and ETB-R is in the endotheliocyte. ET-1 is the most abundant isoform and the best characterized in vivo one of the two receptors. Bosentan is a nonselective dual endothelin-receptor antagonist, and it is widely used to treat PH. It can improve the exercise capacity and hemodynamics of PH patients.

In recent years, bosentan has been shown to have some function in acute lung injury and PF by three methods, which included reducing the right ventricular systolic pressure, decreasing reactive oxygen species release and relieving the inflammatory reaction [17] [24] [25]. Bosentan therapy has shown to increase susceptibility to infection [26], but during our experiment, no symptoms of infection were observed.

In our research, the ET-1 and TGF-β1 content of rat serum and lungs significantly increased following intragastric administration of PQ, and the HYP of the lung tissue increased more than the control group. In comparison to the PQ group, early treatment with bosentan significantly reduced the ET-1,TGF-β1 and HYP content in the serum and lungs on the 21st day. The dynamic changes in these data are consistent with the histomorphological changes. This trend indicates that early treatment with bosentan after PQ poisoning may be helpful in ameliorating lung injury and decreasing lung fibrosis.

In summary, bosentan had potentially protective effects in the lungs of the rat model of acute PQ poisoning, possibly through anti-cytokine and anti-fibrosis action. However, more studies are needed to further verify its effectiveness and to confirm its clinical benefits.
